# Diffusion tensor imaging of the structural integrity of white matter correlates with impulsivity in adolescents with internet gaming disorder

**DOI:** 10.1002/brb3.753

**Published:** 2017-06-21

**Authors:** Xin Du, Linlin Liu, Yongxin Yang, Xin Qi, Peihong Gao, Yang Zhang, Jiyu Zhu, Guijin Du, Shouping Dai, Xiaodong Li, Quan Zhang

**Affiliations:** ^1^ Department of Radiology and Tianjin Key Laboratory of Functional Imaging Tianjin Medical University General Hospital Tianjin China; ^2^ Department of Psychology Linyi Fourth People's Hospital Linyi Shandong China; ^3^ Department of Radiology Linyi People's Hospital Linyi Shandong China

**Keywords:** diffusion tensor imaging, impulsivity, internet gaming disorder, white matter

## Abstract

**Introduction:**

Internet gaming disorder (IGD) is usually defined as the inability of an individual to control internet gaming resulting in serious negative consequences, and trait impulsivity has been viewed as a hallmark feature of IGD. Recent studies have suggested that the structural integrity of the white matter (WM) plays an important role in the neuromediation of an individual's impulsivity. However, no study has examined the association between WM integrity and impulsivity in IGD adolescents.

**Methods:**

In this study, 33 adolescents with IGD and 32 healthy controls (HCs) were recruited, and the intergroup differences in the relationships between impulsivity and fractional anisotropy (FA) values across the whole brain WM were investigated using voxel‐wise correlation analyses.

**Results:**

Our results revealed significant intergroup differences in the correlations between impulsivity and the FA values of the right corticospinal tract (CST) and the right occipital WM. Region of interest‐based tests revealed that the FA values of these clusters were positive or insignificantly correlated with impulsivity in the IGD adolescents contrasted to the significantly negative correlation in the HCs.

**Conclusions:**

This altered correlations in the IGD adolescents might reflect potential WM microstructural changes which may be associated with the greater impulsivity of IGD adolescents and provide possible therapeutic targets for interventions in this population.

## 1 INTRODUCTION

Internet gaming disorder (IGD) is the most prevalent form of internet addiction in Asia (e.g., China and Korea) (Dong, Devito, Du, & Cui, 2012) and is defined as an individual's inability to control internet gaming resulting in negative consequences, such as psychological, social, school, and/or work difficulties in one's life (Cao, Su, Liu, & Gao, 2007; Young, 1998). In recent years, and of significant public importance, IGD was classified into section III, that is, conditions for future study, of the Diagnostic and Statistical Manual of Mental Disorders, Fifth Edition (DSM‐5) (Association AP, 2013). Moreover, impulsivity has been demonstrated to play an important role in the development and progression of IGD. Some researchers (Cao et al., 2007; Shapira, Goldsmith, Keck, Khosla, & McElroy, 2000; Young, 1998) have suggested that internet addiction, including IGD, was an impulse disorder or was at least related to impulse control. Recent studies (Cao et al., 2007; Chen et al., 2015; Ko et al., 2014, 2015; Luijten, Meerkerk, Franken, van de Wetering, & Schoenmakers, 2015) have found that adolescents with IGD/internet addiction had greater impulsivity compared to that of healthy controls (HCs). Behavioral studies using impulse control‐related tasks (e.g., Go–NoGo, Go–Stop, and/or Stroop paradigms) have demonstrated behavioral control difficulties in IGD adolescents (Cao et al., 2007; Dong, Zhou, & Zhao, 2010, 2011; Lin et al., 2012; Liu et al., 2014; Luijten et al., 2015). In a prospective longitudinal investigation, Gentile (Gentile et al., 2011) revealed that impulsivity was a risk factor for the development of IGD. Moreover, impulsivity and selective attention have been reportedly involved in the pathogenesis of IGD, as well as the severity of IGD in a study about the medication treatment of IGD (Song et al., 2016). Given that great impulsivity is a potential cause of dangerous behaviors (e.g., suicide attempts and crime) in adolescents, investigations into the neural substrates of the greater impulsivity of IGD adolescents are expected.

Many studies have revealed significant correlations between impulsivity and structures or functions of multiple gray matter regions in healthy subjects (Boes et al., 2009; Brown, Manuck, Flory, & Hariri, 2006; Cho et al., 2013; Dambacher et al., 2015; Farr, Hu, Zhang, & Li, 2012; Gardini, Cloninger, & Venneri, 2009; Matsuo et al., 2009; Muhlert & Lawrence, 2015; Schilling et al., 2012, 2013, 2013; Van den Bos, Rodriguez, Schweitzer, & McClure, 2015). In the past few years, diffusion tensor imaging (DTI) technique shows great promise to evaluate the integrity of white matter (WM) tracts in human brain (Guo et al., 2012, 2012), and the white matter (WM) integrity of the bilateral frontal and temporal lobes was negatively associated with impulsivity in healthy adolescents (Olson et al., 2009). Addiction‐related studies have also revealed significant correlations between greater impulsivity and the integrity of many WM regions. For example, Herting, Schwartz, Mitchell, & Nagel (2010) reported a relationship of the FA values in the left inferior longitudinal fasciculus and the right optic radiation with greater impulsivity as detected with a delay discounting task in youth with family histories of alcohol abuse, which suggests that disrupted white matter microstructure may act as an intrinsic risk factor for alcohol use disorder. A study by Fortier et al. (2014) found that decreased FA values throughout the fronto‐striatal circuits may mediate impulsive behavior in abstinent alcoholics. Additionally, a relationship between WM integrity and drug abuse has also been demonstrated. Negative correlations between greater impulsivity and the FA values of the anterior corpus callosum and frontal WM have been found in cocaine abusers (Moeller et al., 2005; Romero, Asensio, Palau, Sanchez, & Romero, 2010). These results indicate that the disrupted integrity of multiple WM regions plays an important role in mediating greater impulsivity in addictive conditions.

Accumulating neuroimaging studies have indicated the neural substrates of the greater impulsivity of IGD adolescents. Recently, functional neuroimaging studies revealed that IGD adolescents exhibit aberrant activations in the fronto‐striatal network, the supplemental motor area, the cingulate cortex, the insula, and the parietal lobes during the performance of impulse control‐related tasks compared with HCs (Chen et al., 2015; Ding et al., 2014; Dong et al., 2012; Ko et al., 2014; Liu et al., 2014; Luijten et al., 2015). Moreover, aberrant effective connectivity in the response inhibition network (Li et al., 2014) and altered resting‐state functional connectivity between multiple brain regions (Kim et al., 2015; Ko et al., 2015) have also been revealed to be correlated with impulsivity in IGD adolescents. Additionally, our previous study of the structural correlates of impulsivity revealed that IGD adolescents exhibited decreased correlations between impulsivity and gray matter volumes in brain areas involved in behavioral inhibition, attention, and emotion regulation compared with HCs (Du et al., 2016). Although DTI studies have demonstrated WM integrity impairments in IGD adolescents compared with HCs (Dong, DeVito, Huang, & Du, 2012; Jeong, Han, Kim, Lee, & Renshaw, 2016; Lin et al., 2012; Weng et al., 2013; Xing et al., 2014; Yuan et al., 2011, 2016), the relationship between impulsivity and WM integrity in IGD adolescents is largely unknown. The previous studies revealed that behavioral addiction is similar to substance addiction in terms of neuropsychology and neurophysiology (Alavi et al., 2012). Therefore, we postulated that IGD, as a behavioral addiction, might also lead to the altered relationships between impulsivity and WM integrity as observed in other addictions (Fortier et al., 2014; Moeller et al., 2005; Romero et al., 2010).

In this study, we aimed to evaluate the relationship between impulsivity and WM integrity based on DTI analysis in a cohort of IGD adolescents relative to demographically matched HCs. Based on previous studies we hypothesized that the HCs with better impulsivity control have greater WM integrity (negative correlation), however, because of IGD adolescents’ characteristics of greater impulsivity, WM integrity of the IGD adolescent would compensatory increase (altered to the positive correlation). This study may bring new insight into the neurobiological presentation of impulsivity in IGD adolescents.

## 2 MATERIALS AND METHODS

### 2.1 Subjects

Thirty‐three male adolescents with IGD were recruited from April to December 2014 from the psychological rehabilitation center of Linyi Forth People's Hospital, and thirty‐two age‐ and education‐matched male HCs were included in our study. All subjects were right handed. The adolescents who answered the Young Diagnostic Questionnaire for Internet addition with five or more “yes” responses were diagnosed with IGD (Young, 1998). Additionally, all of the IGD adolescents in this study were required to meet two additional inclusion criteria, that is, an online game playing time of ≥4 hr/day and a Young's internet addiction test (IAT) score ≥ 50. None of the HCs in our study reached the diagnostic criterion of the Young's Diagnostic Questionnaire for internet addition, spent no more than 2 hrs/day on online game playing, and had an IAT score of less than 50. The exclusion criteria for all subjects were the following: (1) any DSM‐IV Axis I diagnosis based on the MINI‐International Neuropsychiatric Interview (MINI), (2) the existence of neurologic disease or neurologic sequelae as assessed with clinical evaluations and medical records, or (3) psychotropic medication use or drug abuse. Additionally, the questionnaire was used to record cigarette and alcohol consumption. Anxiety and depression states were assessed using the Self‐Rating Anxiety Scale (SAS) and the Self‐Rating Depression Scale (SDS). A battery of neuropsychological tests was performed to assess the participants’ cognitive domains. The Intelligence Quotients (IQs) of all participants were examined by using standard Rawen's progressive matrices. Working memory was assessed with the forward and backward digit span test, and short‐ and long‐term memories were tested using the Auditory Verbal Leaning Test. Information processing speed was tested with the trail‐making test (TMT‐A). Execute function was tested with the TMT‐B. The protocol of this study was approved by the Ethical Committee of Tianjin Medical University General Hospital, and all of the participants and their guardians provided written informed consent according to institutional guidelines.

### 2.2 Impulsivity assessment

The Barratt Impulsiveness Scale 11 (BIS‐11) (Patton, Stanford, & Barratt, 1995) was used to assess the impulsivities of all subjects in this study. The BIS‐11 is a self‐report measure designed to assess impulsivity that consists of 30 items and includes the following three subscales: Attentional Impulsiveness (AI, attention deficits, rapid thoughts, and lack of cognitive patience), Motor Impulsiveness (MI, impetuous action), and Nonplanning Impulsiveness (NI, lack of future orientation). All items were answered on a 4‐point Likert scale (rarely/never, occasionally, often, and almost always/always). The sum of the three subscales scores was taken as the Raw Impulsiveness (RI). Higher scores reflect greater levels of impulsivity.

### 2.3 Data acquisition

DTI data were acquired using a Siemens 3.0‐T scanner (Magnetom Verio, Siemens, Erlangen, Germany) with single‐shot spin‐echo echo planar imaging sequence and the following parameters: TR = 7000 ms, TE = 95 ms, flip angle = 90°, FOV = 256 mm × 256 mm, matrix size = 128 × 128, slice thickness = 3 mm, 48 slices with no gap, 64 encoding diffusion directions with a b value of 1,000 s/mm^2^, and one volume was also acquired without diffusion weighting (b = 0 s/mm^2^). T1‐weighted volumetric magnetization‐prepared rapid gradient‐echo sequence was used to acquire a series of 192 contiguous sagittal high‐resolution anatomical images with the following parameters: TR = 2,000 ms, TE = 2.34 ms, TI = 900 ms, flip angle = 9°, FOV = 256 mm × 256 mm, slice thickness = 1 mm, and matrix size = 256 × 256.

### 2.4 DTI data processing

DTI preprocessing was performed using FMRIB's diffusion toolbox (FSL 4.0, http://www.fmrib.ox.ac.uk/fsl) and comprised the following steps: eddy current distortions and head motion artifacts in all DTI data were corrected by applying the affine alignment of each diffusion‐weighted image to the nondiffusion image; the skull was stripped from each participant's DTI images using the robust brain extraction tool (BET); and FA, radial diffusivity (RD), and axial diffusivity (AD) maps were then calculated using the FMRIB diffusion toolbox in FSL. The individual diffusion indices (FA, RD, and AD) were coregistered into MNI space using two‐step method. First, the brain‐extracted b = 0 images of each subject were coregistered with his T1 images using an affine method (12 parameters); then, the T1 images were affinely coregistered into the T1 template of MNI space; finally, the diffusion indices were written into MNI space using the affine parameters generated from the above steps and were resliced into 2 × 2 ×  2 mm^3^. The normalized FA, RD, and AD maps were smoothed with an isotropic Gaussian kernel of 6‐mm full width at half maximum.

### 2.5 Statistical analysis

Two‐sample *t*‐tests were used to examine the intergroup differences in age, education, online game playing time (hrs/day), IAT score, SAS score, SDS score, BIS‐11 scores, and cognitive variables using SPSS 18.0. Chi‐square test was used to examine the intergroup differences in smoking rate. The significance level was set at *p *<* *.05.

Voxel‐wise statistical analysis of the correlations between impulsivity and FA values was performed using FSL's permutation‐based nonparametric testing with 5,000 random permutations. The FA values were considered as dependent variables, group (HCs vs. IGD), BIS‐11 (RI, AI, MI, and NI) scores, and their interactions were considered as interesting independent variables, and age, SAS score, and SDS score were treated as confounding variables. The BIS‐11 (RI, AI, MI, and NI) scores of each subject were demeaned in each group before entry into the model. A priori WM template binarized with a threshold > 0.3 was used as a mask to confine the statistical analysis within the WM regions. First, the correlations between impulsivity and the FA values of each group were estimated by calculating the regression coefficients between the FA value of each voxel within the WM mask and the BIS‐11 (RI, AI, MI, and NI) scores. Next, the intergroup differences in the regression coefficients were compared in the model. Threshold‐free cluster enhancement (TFCE) was used to correct for multiple comparisons (*p* < .05).

The regions with significant intergroup differences in the correlations between the FA values and the BIS‐11 (RI, AI, MI, and NI) scores were defined as the regions of interest (ROIs). The average FA values in the ROIs were then extracted. ROI‐based partial correlation analyses between the average FA values and the corresponding BIS‐11 (RI, AI, MI, and NI) scores were also performed in each group after controlling for age and the SAS and SDS scores to validate the results of the voxel‐wise analyses. The Bonferroni correction was used to control the multiple comparisons.

Voxel‐wise statistical analysis of the intergroup differences in the FA, AD, and RD was performed using FSL's permutation‐based nonparametric testing with 5,000 random permutations. TFCE was used to correct for multiple comparisons (*p* < .05).

## 3 RESULTS

### 3.1 Demographic and clinical data

There were no significant intergroup differences in terms of age, education, cognitive variables, or smoking rate. None of the subjects habitually consumed alcohol. The online game playing time (hrs/day), IAT score, SAS score, SDS score, and BIS‐11 (RI, AI, MI, and NI) scores were significantly higher in the IGD group than those in the HCs. All of the demographic and clinical data are listed in Table 1.

**Table 1:** Demographic and clinical data

Item	IGD *N* = 33	HCs *N* = 32	*Statistics*	*p*
Age (years)	16.82 ± 3.46	17.28 ± 3.01	−0.575	.567
Education (years)	9.70 ± 2.68	11.06 ± 2.87	−1.984	.052
IQ	49.36 ± 6.33	48.88 ± 5.60	0.329	.743
Smoking (yes/no)	8/25	4/28	1.488	.223^a^
Online game playing time (hrs/day)	9.42 ± 4.47	1.33 ± 0.66	10.133	<.001
IAT score	70.00 ± 11.54	32.97 ± 7.60	15.228	<.001
RI score	68.39 ± 12.14	55.53 ± 8.97	4.848	<.001
AI score	15.15 ± 3.95	12.94 ± 2.77	2.611	.011
MI score	21.39 ± 4.09	17.75 ± 3.12	4.027	<.001
NI score	28.79 ± 6.12	22.72 ± 4.15	4.663	<.001
SAS score	43.36 ± 8.72	36.16 ± 6.39	3.791	<.001
SDS score	50.61 ± 11.34	39.78 ± 9.07	4.241	<.001
Short memory	59.12 ± 8.49	59.09 ± 7.83	0.014	.989
Long memory	12.61 ± 2.33	13.22 ± 2.15	−1.100	.275
FDS	9.12 ± 1.45	8.69 ± 1.15	1.333	.187
BDS	6.88 ± 1.75	6.56 ± 1.29	0.828	.411
TMT‐A (s)	31.80 ± 1.07	34.24 ± 11.28	−0.895	.374
TMT‐B (s)	89.71 ± 30.34	89.47 ± 38.10	0.028	.977

AI, Attentional Impulsiveness; BDS, backward digit span test; FDS, forward digit span test; IAT, internet addiction test; IQ, Intelligence Quotient; MI, Motor Impulsiveness; NI, Nonplanning Impulsiveness; RI, Raw Impulsiveness; SAS, Self‐Rating Anxiety Scale; SDS, Self‐Rating Depression Scale; TMT, trail‐making rest.

a Chi‐square test.

### 3.2 Voxel‐wise correlation comparison

**Figure 1:**
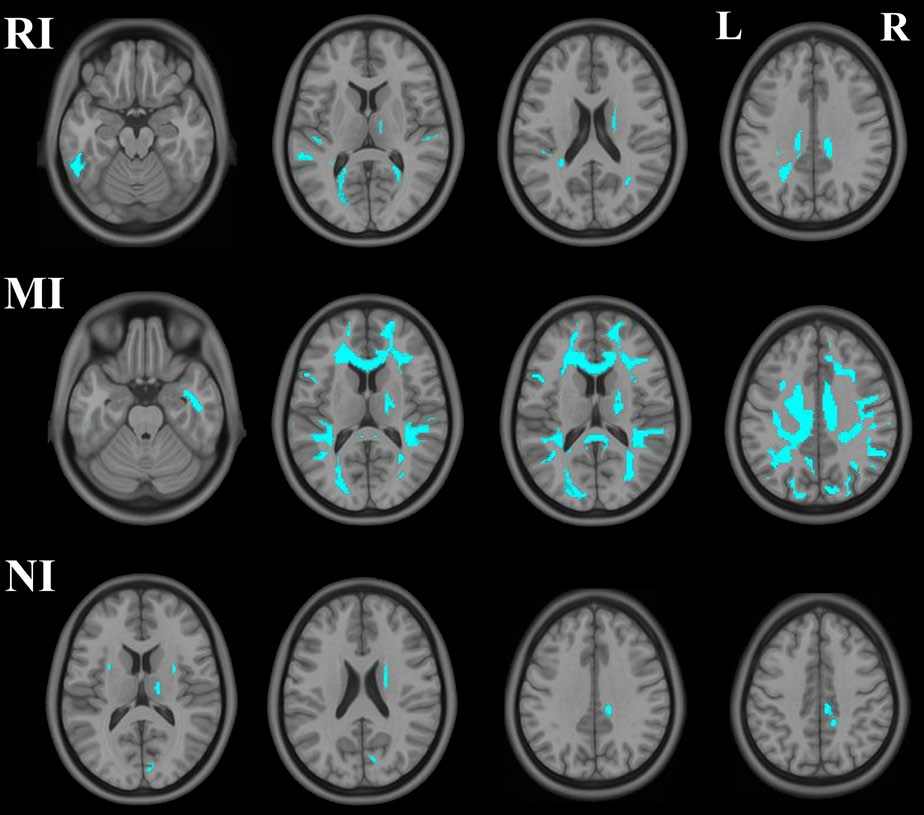
Brain regions showing negative correlations between the FA values and impulsivity (RI, MI, NI) in the HCs

The voxel‐wise correlation analyses revealed that, in the HC group, the RI score was negatively correlated with the FA values of the bilateral temporal, parietal, and occipital WM regions and the right internal capsule. The MI score was negatively correlated with the FA values of the bilateral frontal, temporal, parietal, and occipital WM regions, corpus callosum, and the posterior crus of right internal capsule. The FA values of the bilateral external capsule, the posterior crus of right internal capsule, and the right occipital and parietal WM regions exhibited negative correlations with the NI score (*p *<* *.05, TFCE correction) (Figure 1). There was no significant correlation of the BIS‐11 scores with the FA values across the whole WM in the IGD group.

**Figure 2:**
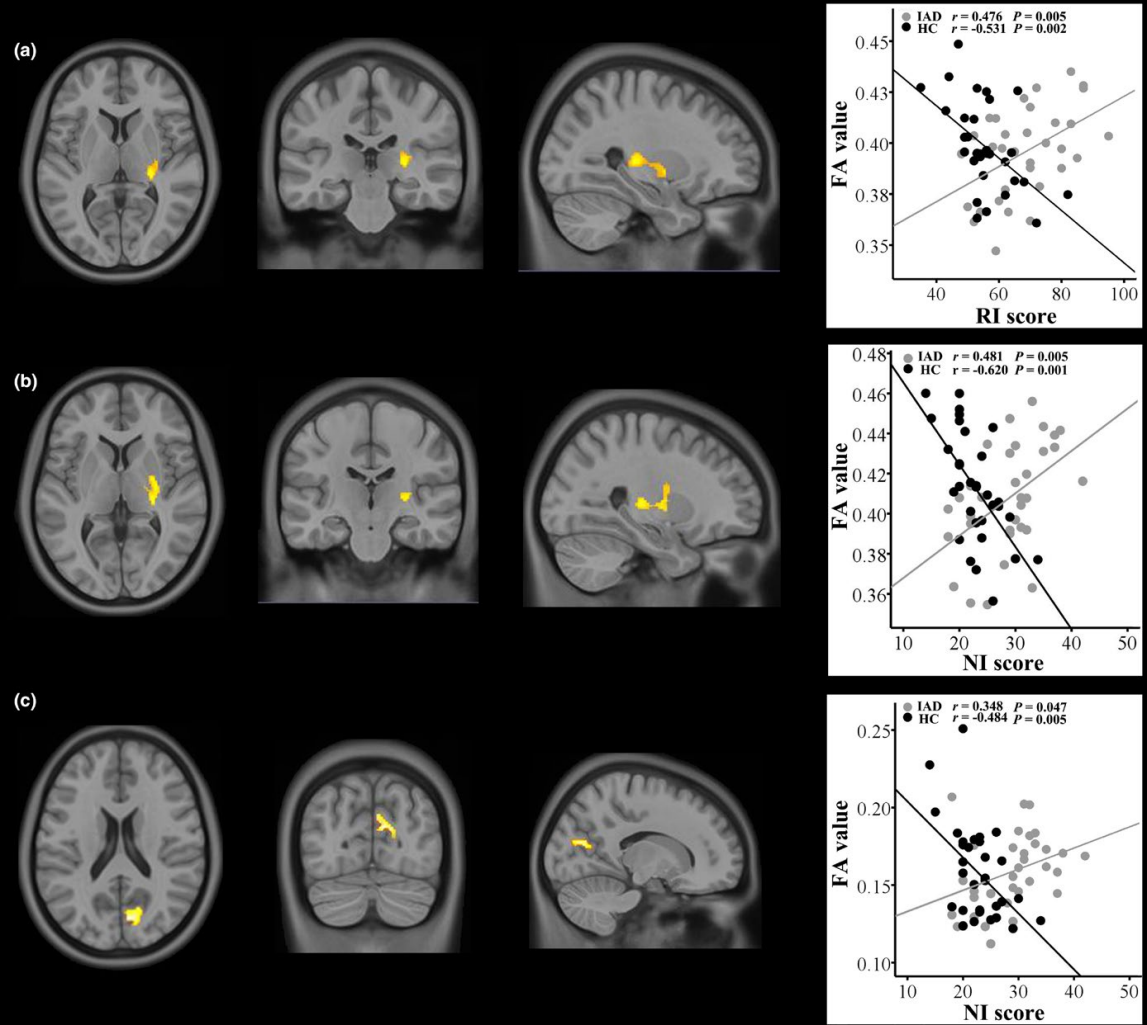
Brain regions showing the altered correlations between the FA values and BIS‐11 (RI and NI) scores in IGD adolescents compared to the HCs. (a), the right CST (at the posterior crus of internal capsule); (b), the right CST (at the posterior crus of internal capsule); (c), the right occipital white matter. Clusters with warm color were the regions with significant intergroup differences in correlation. ROI‐based correlation analyses (right column) show that FA values of the three clusters negatively correlated with RI/NI score in the HCs, while positive or insignificant correlation in the IGD group

The voxel‐wise correlation analyses revealed that, compared with the HCs, the IGD adolescents exhibited a higher correlation between the RI score and the FA values of the right CST (at the posterior crus of the internal capsule). The IGD adolescents also exhibited higher correlations between the NI score and the FA values of the right CST (at the posterior crus of the internal capsule), and between the NI score and the FA value of the right occipital WM region (*p *<* *.05, TFCE correction) (Table 2, Figure 2). There were no significant intergroup differences in the correlations of the AI and MI scores with the FA values across the whole WM.

**Table 2:** Regions showing significant intergroup differences in correlations between the FA values and impulsivity

BIS‐11	Fiber tract	Region	Peak MNI Coordinates			*P* value (Corrected)	Cluster Size (voxels)
			X	Y	Z		
RI	R_CST	Posterior crus of internal capsule	26	−28	4	0.010	222
NI	R_CST	Posterior crus of internal capsule	28	−24	6	0.018	248
		R_Occipital WM	16	−80	−14	0.014	255

BIS‐11, Barratt Impulsiveness Scale 11; CST, corticospinal tract; NI, nonplanning impulsiveness; R, right; RI, raw impulsiveness; WM, white matter.

### 3.3 ROI‐wise correlation analysis

Three clusters with significant intergroup differences in the correlations between the FA values and impulsivity were defined as the ROIs. ROI‐based correlation analyses revealed significant negative correlations between the BIS‐11 (RI and NI) scores and the FA values within three ROIs in the HCs (*p *<* *.05/6, Bonferroni correction), whereas significant positive correlations were observed between the FA values of the right CST and the BIS‐11 (RI and NI) scores in the IGD group (*p *<* *.05/6, Bonferroni correction) (Figure 2). There was no significant correlation between the FA values of the right occipital WM region and the NI scores in the IGD group.

### 3.4 Intergroup comparisons of the FA, RD, and AD values

There were no significant intergroup differences in the FA, RD, or AD values in the voxel‐wise intergroup comparisons across the whole WM.

## 4 DISCUSSION

In this study, altered correlations between WM integrity and impulsivity in the IGD adolescents were evaluated. In the HCs, the FA values of multiple WM regions exhibited negative correlations with impulsivity, which is consistent with the results of a previous study regarding the relationships between white matter integrity and delay discounting behavior in healthy young subjects (Olson et al., 2009). The IGD adolescents exhibited positive or insignificant correlations between impulsivity and the FA values of the right CST and the right occipital WM region contrasted to the significantly negative correlation in the HCs.

The CST contains fibers running from the primary motor, premotor, supplementary motor, somatosensory, parietal, and cingulate cortices to the spine and plays essential roles in transferring motor‐related information, such as voluntary movement and motor control (Porter, 1985). Previous neuroimaging studies have provided evidences that the CST projection regions play important roles in modulating impulsivity in healthy subjects (Brown et al., 2006; Farr et al., 2012). An fMRI study of healthy drinkers revealed that the activation of right frontal motor/premotor region during a response inhibition task was inversely related to impulsivity score, which indicated that the great impulsivity was related to impairment in motor control system (Weafer et al., 2015). A study by Olson et al. (2009) revealed that higher FA values of the right CST were associated with less impulsive performance in the delay discounting task in healthy adolescents. In our study, negative correlations between impulsivity and the FA values of the right CST were found in the HCs, which was consistent with the results of Olson's studies. Kelvin's voxel‐wise correlation analysis also indicated that low FA values of the posterior crus of the internal capsule are associated with increased impulsivity as measured by the BIS‐11 in chronic cocaine users (Lim et al., 2008). These results suggest that the altered correlations between impulsivity and the FA values of the CST in the IGD adolescents might reflect potential WM microstructural changes that may be associated with the greater impulsivity of IGD adolescents.

In our study, the IGD adolescents did not exhibit significant alterations in the FA, AD, or RD values compared with the HCs, but exhibited positive correlations between impulsivity and the FA values contrasted to the significantly negative correlation in the HCs. There are two possible explanations for the altered correlations between impulsivity and the DTI metrics in the IGD adolescents in the absence of DTI metric changes. Genetic factors contribute to the development of IGD (Li, Chen, Li, & Li, 2014). The IGD adolescents enrolled in our study were still in the process of WM maturation, and different genetic backgrounds might have caused them to undergo WM development and plasticity in different manners relative to the healthy subjects (Giedd & Rapoport, 2010). Therefore, different genetic backgrounds may be partly responsible for the altered correlations between impulsivity and the DTI metrics in the IGD adolescents. However, this explanation requires confirmation with genetic studies in the future. Another possible explanation for the altered correlations between impulsivity and the DTI metrics in the IGD adolescents is related to the effect of IGD on WM microstructures. Increased WM integrity of the CST in IGD individuals has been demonstrated in previous studies (Jeong et al., 2016; Yuan et al., 2011; Zhang et al., 2015). Although there were no significant intergroup differences in the DTI metrics of the CST, the positive correlations were found between impulsivity and the FA values in the IGD adolescents, which indicate a tendency among the IGD adolescents to have relatively higher FA values for impulsivity inhibition. The IGD adolescents enrolled in our study had no significant alterations in cognitive performances, which suggested that IGD had a subtal effect on their cognitive function at the time of examination, and a longitudinal study is needed to confirm the dynamic effect of IGD on WM microstructures. Additionally, multiple fMRI studies of inhibitory control in IGD adolescents have demonstrated greater impulsivity and lower inhibitory control accompanied by aberrant brain activations in the precentral gyrus and supplemental motor area in IGD adolescents compared with healthy subjects (Chen et al., 2015; Ding et al., 2014; Dong et al., 2012; Liu et al., 2014; Luijten et al., 2015). Taken together, these findings make it plausible to postulate that the functional and structural states of the motor system, including the cortex and the WM fiber tracts, were associated with greater impulsivity in the IGD adolescents.

Additionally, in contrast to the HCs, the correlations between impulsivity and the FA values of the right occipital WM region disappeared in the IGD adolescents in our study. Increased FA values of the occipital WM have been showed in the IGD adolescents, which may arise secondary to repetitive online game play (Jeong et al., 2016). Gray matter volume within the occipital cortex was positively correlated with the video gaming addiction score and the amount of lifetime video gaming (Kuhn & Gallinat, 2014). Also, riskier performance in the Iowa Gambling Task was related to reduced occipital WM integrity in alcohol‐dependent subjects (Zorlu et al., 2013). It is plausible to postulate that, as a visual information transferring tract, the right occipital subcortical WM may have potential microstructural changes in the IGD adolescents which contributed to the altered correlation between impulsivity and the FA values.

Some limitations of this study should also be noted. First, the cross‐sectional design of our study precluded us from drawing conclusions about the causal relationship between the absent correlations and IGD. To address whether the absent correlations in the IGD adolescents are due to preexisting abnormal structural development or secondary to the IGD, genetic studies and longitudinal studies are warranted. Second, only male adolescents were included in our study because of the substantially greater prevalence of IGD in young males relative to women and other age groups. Our findings should be considered to be specific to male adolescents with IGD. Lastly, classification of IGD that was solely based on the self‐report measures (YDQ and IAT) which is not appropriate enough, more detailed clinical interviews should be included in assess IGD in the future research.

In conclusion, the negative correlations between impulsivity and the FA values within multiple WM regions in the HCs indicated a normal neuromechanism of impulse control in the healthy subjects. The altered correlations between impulsivity and the FA values of the CST and the occipital WM in the IGD adolescents might reflect potential WM microstructural changes that may be associated with the greater impulsivity of IGD adolescents. Impulsivity and selective attention have been reportedly involved in the pathogenesis of IGD and related to the severity of IGD in a study on the medication treatment of IGD (Song et al., 2016). Our study further defined neurobiological signatures for impulsivity in the IGD adolescents, and implicated that the treatment targeted on improving altered correlation between the impulsivity and WM integrity would warrant additional investigation.

## CONFLICT OF INTEREST

None declared.
